# Body-Posture Recognition by Undergraduate Students Majoring in Physical Education and Other Disciplines

**DOI:** 10.3389/fpsyg.2020.505543

**Published:** 2020-09-16

**Authors:** Weidong Tao, Bixuan Du, Bing Li, Weiqi He, Hong-Jin Sun

**Affiliations:** ^1^Department of Psychology, School of Teacher Education, Huzhou University, Huzhou, China; ^2^Research Center of Brain and Cognitive Neuroscience, Liaoning Normal University, Dalian, China; ^3^School of Foreign Languages, Huzhou University, Huzhou, China; ^4^Department of Psychology, Neuroscience & Behaviour, McMaster University, Hamilton, ON, Canada

**Keywords:** body posture recognition, configural processing, inversion effect, expertise recognition, face specific processing

## Abstract

Humans are more proficient at processing visual display of body posture when the body is in upright orientation, compared to when inverted (inversion effect). Here we investigated whether extensive exposure or expertise on body posture recognition would affect the efficiency with which body-posture is processed. Using whole-body and piecemeal-body postures as stimuli, we performed two experiments to investigate whether body-posture recognition differed between two groups of participants: undergraduates majoring in physical education (PE) and those in other subjects (non-PE), respectively. These two groups differed significantly in the frequency and intensity of exercise per day and/or accumulated exercise time. In our experiments, following initial presentation of an image of a body posture, participants were shown the same or a different stimulus and were asked to report whether or not they had been previously shown the same image. The orientations of the body postures were also varied between trials. Our results showed that, in Experiment 1, for whole-body posture recognition, both the PE and non-PE groups showed a robust body-inversion effect in terms of both error rate and reaction time (RT), but the magnitude of the body-inversion effect in the RT measure was greater in the PE than the non-PE group. In Experiment 2, for piecemeal-body postures, both groups showed the inversion effect in terms of both error rate and RT measures and the PE group made fewer overall errors than the non-PE group. These cumulative results suggest that a superiority effect exists for PE participants compared with non-PE participants. Our results are generally consistent with the expertise hypothesis.

## Introduction

Humans are exposed to various types of objects in their daily lives. When certain stimuli are repeatedly encountered, people obtain extensive visual experiences of recognizing such stimuli and consequently develop more efficient approaches in processing and representing them in their brains. For instance, during social interaction, faces in an upright orientation are repeatedly processed; thus, upright faces are more familiar to people than inverted faces. Yin (1969) used the “inverted paradigm” to compare recognition performance for faces with that for other objects (e.g., houses). The “inverted paradigm” consists of presentation of upright or inverted images of faces or other objects (e.g., airplanes, houses, etc.). Following initial presentation of a stimulus image, participants were subsequently shown the same or a different stimulus and were asked to report whether or not they had been previously shown that same image. Findings from this paradigm illustrate that the inversion of faces disproportionately impairs recognition in comparison to the inversion of non-face objects such as airplanes. These findings suggest that faces are processed in the configural manner (Carey, 1992; Maurer et al., 2002; Van Belle et al., 2010a). That is, in order to efficiently recognize faces, one must be able to process the relative spatial relations between different facial components (e.g., nose, mouth, eyes, etc.). When faces are inverted, the ability to process such configural information becomes impaired. Research has suggested that configurational processing appears to have been specifically adopted for the processing of faces. A considerable number of behavioral and neurophysiological studies have supported the hypothesis that humans have specialized cognitive and neural mechanisms for processing faces, known as the “face-domain-specific hypothesis.”

However, the face-domain-specific hypothesis has been challenged by some researchers. Diamond and Carey (1986) found that individuals who had high levels of exposure to dogs (i.e., dog experts) demonstrated the inversion effect upon being asked to recognize upright and inverted images of dogs, but such an effect was not present in people with low levels of exposure to dogs. Campbell and Tanaka (2018) found that budgerigar experts showed equal recognition deficits for inverted faces and inverted budgerigars, but budgerigar novices showed a significant inversion effect for faces only, not for budgerigars. Similarly, the inversion effect has been observed in participants who received extensive training in discriminating artificial novel objects. For example, Gauthier and Tarr (1997) created a set of novel objects called “Greebles” and used them as stimuli to test recognition performance among trained and untrained novices. Their findings demonstrated that it took longer for trained participants (i.e., experts) to recognize transformed Greebles (i.e., Greebles with a modified appearance) than studied Greebles, and it was merely so when the Greebles were presented in upright orientation. Further, using pictures of houses, Husk et al. (2007) also found the inversion effect among participants who had been trained to recognize such images. Gauthier et al. (1999) proposed the “expertise” hypothesis, a domain-general mechanism. That is the mechanisms used for face-processing are also involved in processing non-face objects for which we have acquired significant visual expertise.

Previous studies have found an inversion effect for body-posture recognition; specifically, upright body-posture recognition has been found to be processed faster and more accurately than recognition of inverted body postures. These findings suggest that body postures may be processed in the configural manner, similar to face-processing, and that when body posture is inverted, participants exhibit impairments in processing such configural information (Reed et al., 2003). Research has demonstrated that these can be seen for a variety of different types of body postures (Brandman and Yovel, 2010; Tao et al., 2014; Arizpe et al., 2017). Electrophysiological studies demonstrate that, when body postures are inverted, the N170 component is delayed and its magnitude is enhanced (Minnebusch and Daum, 2009; Tao et al., 2014). In addition, to identify the specific part of the body that contributes to the inversion effect, studies have found that the body-inversion effect is eliminated when the head is removed from body-posture stimuli (Brandman and Yovel, 2010; Mohamed et al., 2011). However, other studies have reported that piecemeal-body postures (removing the head and trunk) could still elicit a robust body inversion effect, although the magnitude of the body inversion effect was reduced (Tao et al., 2014).

To date, few studies have investigated whether expertise would influence recognition for body postures. Previous studies have revealed a positive correlation between the extent of physical training and cognitive function (Hillman et al., 2008; Di Russo et al., 2010; Alves et al., 2013; Pacesova et al., 2018). Further, athletes have been shown to perform better in domain-general cognitive tasks than non-athletes (Voss et al., 2010; Montuori et al., 2019). In a previous study using point-light displays as stimuli and a sample comprising expert female ballet dancers and non-expert controls, Calvo-Merino et al. (2010) reported an effect of expertise on the body inversion effect. However, there is a lack of research regarding the sensitivity of athletes to body-posture recognition and the associated inversion effect. Expertise in body posture can typically be seen within athlete populations, due to their experience with alternate body postures seen during ball games, gymnastics, and so on. In addition to professional athletes, undergraduate students majoring in physical education (PE) could also exhibit a high level of expertise on body posture recognition than those majoring in other subjects (non-PE). After all, PE undergraduates should have devoted more time to observing body postures, and their training likely demands a high level of performance in body posture processing, thus leading to greater perceptual learning for various body postures. If the object expertise hypothesis can be held true, PE students should demonstrate a larger inversion effect in body-posture recognition relative to non-PE subjects. However, it must also be acknowledged that although PE students likely have greater exposure (in both time and quality) to body posture, the body is a form of social stimulus in everyday life; even for individuals who are not extensively exposed to athletics-related stimuli.

In the present study, we conducted two experiments, both involving a group of PE undergraduates and a group of non-PE undergraduates (participant samples differed for the two experiments). In Experiment 1, both groups performed a whole-body posture recognition task, while in Experiment 2, they performed a piecemeal-body posture recognition task. The main purpose of Experiment 1 was to investigate whether there were any differences in performance between PE and non-PE undergraduates for whole-body posture recognition and the associated inversion effect. If frequent exercise and longer time spent in exercising can enhance body-posture recognition, then the performance of the PE undergraduates in this task should be better than that of the non-PE undergraduates. We also predicted that both groups would show a significant body inversion effect, because previous studies have reported that inverted whole-body postures induce impaired performance in a general population (Tao and Sun, 2013; Tao et al., 2014). In Experiment 2, we extended our investigation by examining whether piecemeal-body postures (with removal of the head and trunk from the body posture) would also elicit a robust body inversion effect for both PE and non-PE undergraduates. If the PE students’ exercise training facilitates their processing of body posture, we hypothesize that there should be a significant difference between the two groups’ respective performances during this task.

## Experiment 1: Whole-Body Posture Recognition for PE and Non-PE Undergraduates

### Materials and Methods

#### Participants

Thirty PE undergraduates (25 men, 5 women, with a mean age of 21.73 years) and 30 non-PE undergraduates (25 men, 5 women, with a mean age of 20.13 years), both recruited from Lingnan Normal University, participated in this experiment. All participants were right-handed and had normal or corrected-to-normal vision. The participants were given 10 yuan in RMB as a reward for completing the experiment. The experiment was conducted in accordance with the regulations of Lingnan Normal University’s institutional research ethics board. Before the experiment, participants were required to self-report the amount of exercise they performed per day (in minutes) and the number of years they had been exercising regularly. These self-reported data showed that the PE undergraduates (*M* = 2.77 h) spent significantly more time exercising per day than the non-PE undergraduates (*M* = 1.28 h), *t*(58) = 5.418, *p* < 0.001. Additionally, the PE students had also been exercising regularly for significantly more years than the non-PE students (*M* = 6.17 and 3.38 years, respectively), *t*(58) = 2.99, *p* = 0.004.

#### Stimuli and Apparatus

**FIGURE 1:**
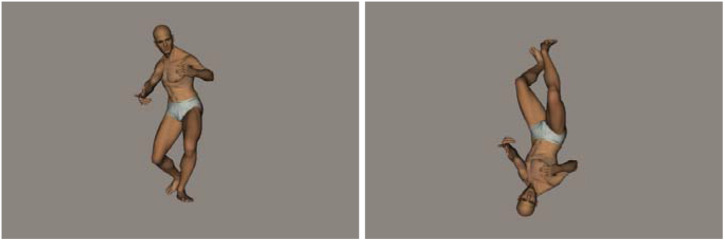
Examples of upright and inverted whole-body postures. Note that, for a given trial, the orientation of the two stimuli was always the same in the actual experiment.

Following the method in Tao and Sun (2013), 20 whole-body postures of a male model were used as stimuli (see Figure 1). Within each trial, two pictures of body postures were presented in sequence, of which there were two types of pairs: identical-body pairs and different-body pairs. Further, between trials, two types of orientations for the body posture were presented: upright and inverted (but the two stimuli in the same trial were always presented in the same orientation). In the different-body-pair trials, the first body-posture stimulus was followed by a mismatched stimulus. The mismatched body-posture stimulus was created by altering two joints of the first body posture, and this served as a “distracter.” In the identical-body-pair trials, the second stimulus was a copy of the body posture presented immediately before. All 20 body postures were presented in both the matched and mismatched formats, with either an upright or inverted orientation.

#### Experimental Design

This experiment adopted a 2 (group: PE and non-PE) × 2 (orientation: upright and inverted) design. The testing comprised three blocks of 40 trials each with 120 trials in total. Twenty practice trials were conducted before the formal experiment.

#### Experimental Procedure

**FIGURE 2:**
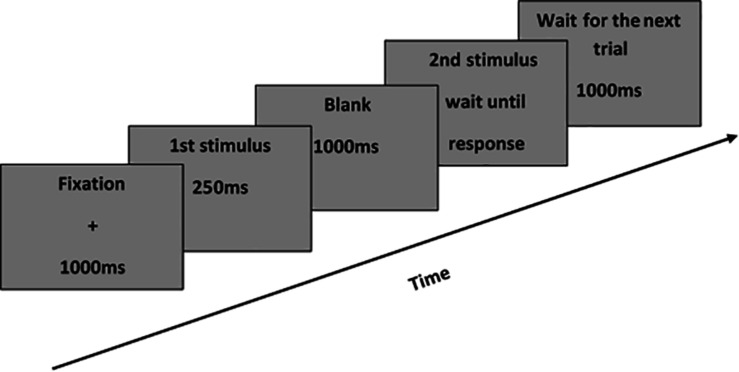
The time sequence of events in a trial.

The experimental trials were compiled using Eprime-1.1 software. A 17-inch computer screen with a resolution of 1024 pixels × 768 pixels was used in the experiment. Each participant was seated at a distance of 60 cm from the computer screen, and the height of the chair was adjusted to ensure that the center of the computer screen was at eye level. In each trial, a black fixation “+” was displayed for 1000 ms. This was followed by the presentation of the first stimulus (for 250 ms), followed by a blank screen (for 1000 ms). After this, the second picture of a body posture, either identical or different from the first picture (but always in the same orientation as the first stimulus), was presented until the participant made a response. After the response as made, a waiting screen was shown for 1000 ms. A graphical scheme of the sequence of events in the trials is shown in Figure 2. Participants were required to press the “1” key, using their right index finger, to indicate that the two body postures were the same, and to press the “2” key, using their right middle finger, to indicate that the two body postures were different. For all trials, participants were asked to respond as quickly and accurately as possible. Both reaction times (RTs) and error rates were recorded.

#### Data Analysis

Following the classical body-posture recognition study (Reed et al., 2003), data for the different-body pairs were removed, and only trials comprising identical-body pairs were analyzed. Further, for RT, only correct responses were included for analysis. Trials with RTs shorter than 200 ms or RTs longer than two standard deviations from the mean were removed. As a result, 1.1% of trials were removed. The Pearson correlation between error rate and RTs showed that *r* = 0.157 and *p* = 0.087, which suggested that there was no significant speed-accuracy tradeoff.

### Results and Discussion

#### Error Rate

**FIGURE 3:**
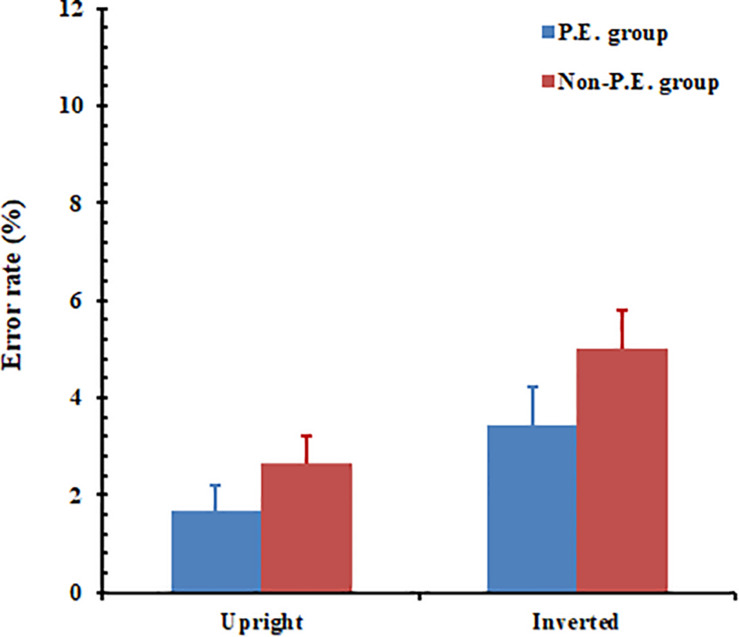
Physical education (PE) and non-PE groups’ respective error data for whole-body posture recognition. Error bars represent standard error of the mean.

The error rate was analyzed using a 2 (orientation: upright, inverted) × 2 (group: PE undergraduates vs. non-PE undergraduates) mixed analysis of variance (ANOVA). The results (see Figure 3) showed that the main effect of orientation was significant, *F*(1, 58) = 10.36, *p* = 0.002. The recognition error rate for the upright body postures (*M* = 2.2%, SE = 0.004) was lower than that for the inverted body postures (*M* = 4.2%, SE = 0.006). The main effect of group was close to significant level, *F*(1, 58) = 3.24, *p* = 0.077. The PE group (*M* = 2.6%, SE = 0.005) performed better than did the non-PE group (*M* = 3.8%, SE = 0.005); however, the interaction between orientation and group failed to reach significance, *F*(1, 58) = 0.19, *p* = 0.665.

#### Reaction Time

**FIGURE 4:**
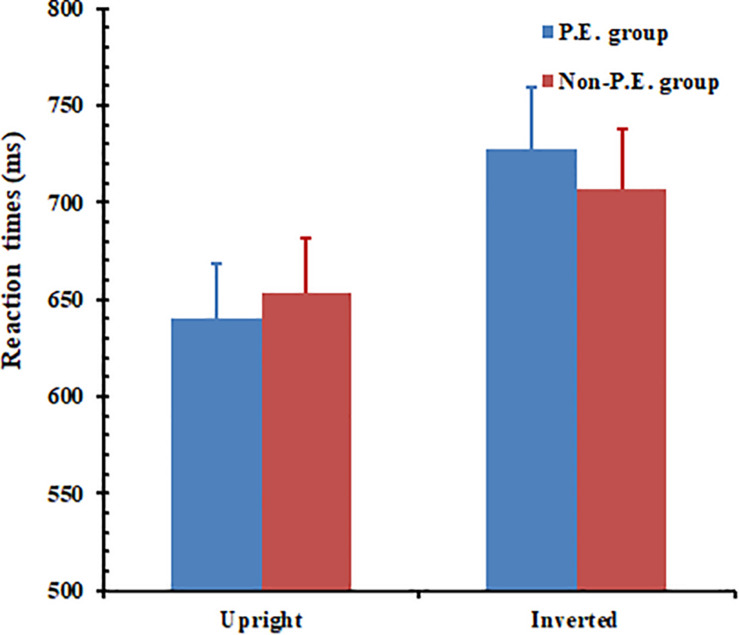
Physical education (PE) and non-PE groups’ respective reaction times (RTs) for whole-body posture recognition. Error bars represent standard error of the mean.

A mixed ANOVA was conducted for RT, and the results (see Figure 4) showed that the main effect of orientation was significant, *F*(1, 58) = 70.04, *p* < 0.001. RT for the upright body postures (*M* = 646 ms, SE = 20.024) was shorter than that for the inverted body postures (*M* = 717 ms, SE = 22.190). No group effect was found, *F*(1, 58) = 0.01, *p* = 0.925. The interaction between group and orientation reached significance, *F*(1, 58) = 4.17, *p* = 0.047. The results of the simple effects analysis showed that the PE group responded in a significantly shorter time to the upright body postures (*M* = 640 ms) than to the inverted body postures (*M* = 727 ms), *F*(1, 58) = 59.08, *p* < 0.01. The same pattern of results were found in the non-PE group *F*(1,58) = 22.29, *p* < 0.01, which shows that upright body postures (*M* = 653 ms) were reacted to in a shorter time than inverted body postures (*M* = 707 ms). Thus, the results showed a main effect of inversion for both groups. However, the PE group showed a greater inversion effect than the non-PE group (87 ms vs. 54 ms).

Experiment 1 was designed to explore, for both groups, the differences in performance when upright and inverted body postures were used as stimuli. The results showed that, for both groups, recognition of upright body postures was significantly faster and more accurate than was that for inverted body postures. This finding implies that both the PE and non-PE undergraduates adopted configural processing to recognize the upright whole-body postures. These findings are consistent with previous findings (Reed et al., 2003, 2006; Tao et al., 2014), indicating that, similar to facial recognition, there is an inversion effect for body-posture recognition.

There was a significant difference between the two groups regarding the inversion-effect in the RT measure. In other words, the magnitude of the inversion effect on RT was significantly larger for the PE participants than for the non-PE participants. This result was likely due to the PE undergraduates having more experience with (upright) body-posture recognition than the non-PE undergraduates, as the former exercised regularly. Thus, it can be inferred that the PE group had greater expertise in body-posture recognition than the non-PE group.

Although our findings showed that the inversion effect in regard to body-posture recognition was larger in the PE than non-PE group, the effect was still significant in the latter group. This could be because all stimuli used in Experiment 1 comprised whole-body postures for which non-PE group have gained enough expertise through daily exposure.

## Experiment 2: The PE and Non-PE Undergraduates’ Recognition of Piecemeal-Body Postures

The results of Experiment 1 showed that the PE group showed a greater inversion effect for RT measure. In order to further observe the superiority effect associated with the expertise hypothesis, in Experiment 2, we examined whether this effect would also be present for piecemeal-body-posture recognition which allowed us to identify the specific body parts that led to the inversion effect.

### Materials and Methods

#### Participants

A different group of 17 PE undergraduates (10 men, 7 women, with a mean age of 20.8 years) and 17 non-PE undergraduates (10 men, 7 women, with a mean age of 20.46 years) from Lingnan Normal University participated in Experiment 2. All the participants were right-handed and had normal or corrected-to-normal vision. They received 10 Yuan in RMB as a reward for participating in the experiment.

#### Stimuli and Apparatus

**FIGURE 5:**
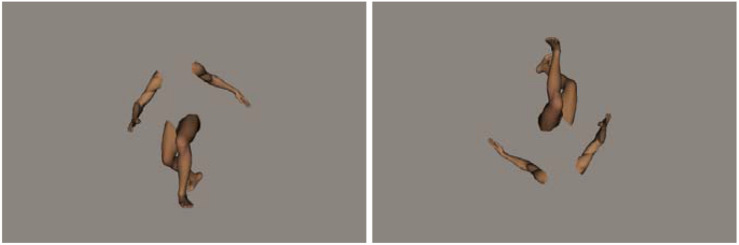
Examples of piecemeal-body posture stimuli. Note that, for a given trial, the orientation of the two stimuli was always the same in the actual experiment.

The stimuli were identical to those used in Experiment 1 except that all body postures were presented with the head and trunk removed (i.e., only the four limbs remained), as shown in Figure 5.

#### Experimental Design

A 2 (group: PE and non-PE) × 2 (orientation: upright body and inverted body) design was adopted in this experiment. The number of trials was identical to that of Experiment 1.

#### Experimental Procedure

The experimental procedure for this experiment was identical to that for Experiment 1.

#### Data Analysis

Results for trials comprising different-body pairs were removed from the data, meaning only the data for the identical-body pairs were analyzed. Only correct responses were included for analysis of RT. Trials with RTs shorter than 200 ms or RTs longer than two standard deviations were removed from the data. As a result, data from 1.5% of trials were removed. The Pearson correlation between error rate and RTs showed that *r* = −0.035 and *p* = 0.78, which suggested that there is no speed-accuracy tradeoff.

### Results and Discussion

#### Error Rate

**FIGURE 6:**
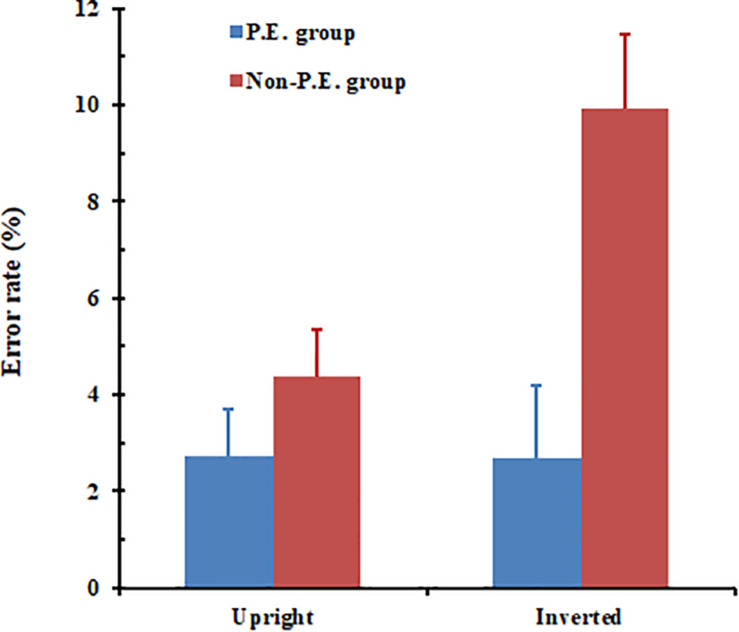
Physical education (PE) and non-PE groups’ respective error data for piecemeal-body posture recognition. Error bars represent standard error of the mean.

A mixed ANOVA with a 2 (orientation: upright and inverted) × 2 (group: PE undergraduates and non-PE undergraduates) factorial design was used. The results (see Figure 6) showed that the main effect of orientation was significant, *F*(1, 32) = 6.19, *p* = 0.018. The results also showed that the error rate regarding recognizing upright body postures (*M* = 3.6%) was significantly lower than that for inverted body postures (*M* = 6.3%). The main effect of group was also significant, *F*(1, 32) = 9.79, *p* = 0.004. The PE group (*M* = 2.7%) performed better than the non-PE group (*M* = 7.2%). The interaction between body-posture orientation and group reached significance, *F*(1, 32) = 6.47, *p* = 0.016. Simple effects analysis showed that, for the PE group, the difference between error rates for upright and inverted body-posture recognition failed to reach a significant level, *F*(1, 32) = 0.000, *p* = 0.969. In contrast, for the non-PE group there was a significant difference between upright and inverted body-posture recognition error rates, *F*(1, 32) = 12.66, *p* = 0.001.

#### Reaction Time

**FIGURE 7:**
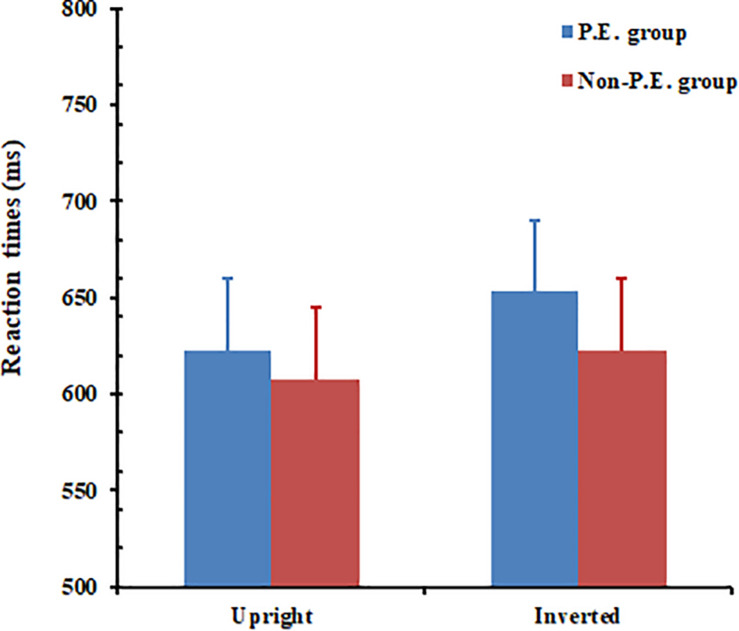
Physical education (PE) and non-PE groups’ respective reaction times (RTs) for piecemeal-body posture recognition. Error bars represent standard error of the mean.

A mixed ANOVA was conducted for RT. The results (see Figure 7) showed that the main effect of orientation was significant, *F*(1, 32) = 6.28, *p* = 0.018. The response time to the upright body postures (*M* = 600 ms) was shorter than that for the inverted body postures (*M* = 624 ms). The main effect of group failed to reach a significant level, *F*(1, 32) = 0.19, *p* = 0.666. The interaction between group and body-posture orientation was not significant, *F*(1, 32) = 0.693, *p* = 0.411. A t-test showed that, for the PE group, the difference between RTs for the upright and inverted body postures reached a significant level, *t*(16) = −2.249, *p* = 0.039. For the non-PE group, there was no significant difference between RTs for the upright and inverted body-postures, *t*(16) = −1.248, *p* = 0.23.

The results of Experiment 2 showed that the PE group performed better than the non-PE group in terms of error rates although not in terms of RTs. In fact, while the PE group generally only made errors in less than 3% of the trials, the non-PE group made errors in about 4 and 10% of the trials for upright and inverted orientations, respectively. The large error for inverted piecemeal body postures in the non-PE group might reflect substantial lack of experience (thus poor perceptual performance) related to those stimuli.

For correct trials, although the inversion effect from RTs reached statistically significance for both groups, the effect was much smaller than that found in Experiment 1. Moreover, for RT measure, there was a lack of significant orientation and group interaction in Experiment 2, unlike the significant interaction observed in Experiment 1. These results suggest that the head and the trunk, as first-order information, play an important role in configural processing. That is, when the head and trunk were removed from the presented body-posture, the inversion effect, although present, became significantly less prominent.

## General Discussion

The current study aimed to investigate whether PE and non-PE undergraduates differ in regard to body-posture recognition. Two major findings came out from these experiments. First, the PE group was generally more accurate (although they did not always show significantly shorter RTs) in recognizing body postures (whole or fragmented pictures) than the non-PE group. Second, there was a significant effect of orientation in both error rate and RT measures (for both whole and fragmented pictures). These findings suggest that body-posture recognition is based on configural processing. Further, the orientation effect revealed in RT measure was greater for the PE group than for the non-PE group and more so for whole body pictures than for fragmented pictures. Thus, our findings from the two experiments demonstrate a general trend wherein PE undergraduates have a stronger expertise-based superiority effect regarding body-posture recognition compared to non-PE undergraduates.

Our findings illustrating a robust inversion effect for whole-body postures and piecemeal-body postures fall in line with previous literature. Reed et al. (2003) previously reported that people are better able to recognize upright body postures than inverted body postures, which suggests that configural processing could not be applied to inverted body postures. These results were further verified by Stekelenburg and de Gelder (2004) who obtained similar findings.

Unlike the whole-body postures, in the piecemeal-body postures, the head and trunk were removed, while the first-order-information regarding the limbs was retained. Brandman and Yovel (2010), by using body-posture stimuli that comprised only the four limbs, reported that the head played a dominate role in the inversion effect in body-posture recognition. However, the present study demonstrated a different finding. The results of Experiment 2 showed that piecemeal-body postures also produced the body-inversion effect; which conforms with Tao et al.’s (2014) findings. These findings also fall in line with the result of another study using biomechanically impossible body postures (in which first-order-information remained, but joints were adjusted), which observed the body-inversion effect in terms of RT (Tao and Sun, 2013). These findings indicate that first-order information, rather than information pertaining to the head, plays an important role in the body inversion effect. Having said that, it is important to note that although a significant inversion effect was found for piecemeal-body postures, the effect (in Experiment 2) was smaller compared to that of the whole body postures (in Experiment 1).

In our study, PE undergraduates showed an expertise-based superiority effect in body-posture recognition compared to non-PE undergraduates. These findings may be explained by the amount of exposure and high demand for perceptual learning of body stimuli. Additionally, these findings are consistent with the views of embodied cognition; that is, that our cognitive processes are affected by bodily experience. In other words, the PE undergraduates’ extensive experience with the visual, kinesthetic, and vestibular consequences of their own movement likely facilitated their body-posture recognition. This prospect is similar to findings demonstrated by Calvo-Merino et al. (2010) who found an expertise effect when analyzing the body-inversion effect among female expert ballet dancers and non-dancer controls. Further, Yu and Zacks (2016) also found that body features enhance the performance of visuospatial transformations. Through a priming paradigm, Daems and Verfaillie (1999) found that stored representations could mediate the identifications of body posture. In addition to the inversion effect, the composite effect of body posture also supports the expertise recognition of body posture (Willems et al., 2014; Vrancken et al., 2017).

Another possible explanation for why PE undergraduates showed an expertise-based superiority effect in regard to body-posture recognition can be found when considering prior findings from examinations of athletes and non-athletes. Regular engagement in sport and exercise improves general cognitive functioning and shapes the functional aspects of the human brain (Kramer and Erickson, 2007; Curlik and Shors, 2013). Previous studies have found that athletes perform better than non-athletes in behavioral tasks targeted at memory, attention allocation, attention flexibility, and executive function (Mann et al., 2007; He et al., 2018; Ishihara et al., 2018). Specifically, meta-analytic reviews have found that sports experts show faster and more accurate performance in regard to spatial memory, visual search tasks, and attentional paradigms (Mann et al., 2007; Voss et al., 2010).

Additional analysis comparing Experiment 1 and Experiment 2 showed that the average RT for whole-body postures in Experiment 1 (682 ms) was significantly longer than that for piecemeal-body postures in Experiment 2 (626 ms). However, such a difference might not be due to group difference in speed-accuracy trade-off. Although the error rate for Experiment 1 (3.19%) was significantly better than that for Experiment 2 (4.94%), the correlation between RT and accuracy was not significant (*r* = 0.039, *p* = 0.597, calculated from combined data from Experiments 1 and 2). Interestingly, in our previous study using within-subject design, we also found that RTs for the whole-body postures was slightly longer than that for the piecemeal-body postures (Tao and Sun, 2013). The RT difference in processing these two types of stimuli may be related to the nature of the difference in processing whole-body postures and piecemeal-body postures. As a previous study discussed, piecemeal-body postures may cause a reversed-body inversion effect in the N170 component in the right hemisphere (Tao et al., 2014). Thus, such postures may need a higher level of attention than whole-body postures. The present study found that both the PE and non-PE groups showed an inversion effect for piecemeal-body posture RT measure, although such results were not found when error rates were examined. This suggests that both experts and non-experts rely on configural information, especially first-order information but the PE group performed better in regard to attention shifting and attention dividing, which helped them recognize the differences between piecemeal-body postures and caused them to make fewer errors. This may explain the PE group’s diminished inversion effect regarding error rate.

Future research may recruit participants with more specific experience. Ishihara et al. (2017) reported that different physical sports place different demands on cognitive functions. Krenn et al. (2018) also stated that the development of executive functions might be influenced by the type of sports engaged in. There is a degree of unilateralism in the present study concerning the classification of participants, even though we strictly chose as PE undergraduates. In addition, a previous study has suggested that a combination of mental and physical training is beneficial for neuronal recruitment (Curlik and Shors, 2013). This will benefit the superiority acquired in the training of the PE group. How exercise benefits the executive functions of children and older adults is a worthwhile future avenue of research.

## Conclusion

In summary, the results of our two experiments showed that PE undergraduates exhibit a superiority effect in response accuracy for both whole-body posture recognition and piecemeal-body posture recognition compared to non-PE undergraduates. PE undergraduates tended to experience a greater inversion effect in RT measure. These superiority effects might be attributed to the PE participants’ extensive prior body-posture experience, which likely facilitated their ability to efficiently process body-posture related stimuli.

## Data Availability Statement

The raw data supporting the conclusions of this article will be made available by the authors, without undue reservation, to any qualified researcher.

## Ethics Statement

The studies involving human participants were reviewed and approved by the IRB School of Teacher Education at Huzhou Normal University. The patients/participants provided their written informed consent to participate in this study.

## Author Contributions

WT contributed to conceptualization and formal analysis. WT and BD contributed to formal analysis. WT, BL, BD, and WH contributed to visualization and writing original draft. H-JS contributed to writing, review, and editing. All authors contributed to the article and approved the submitted version.

## Conflict of Interest

The authors declare that the research was conducted in the absence of any commercial or financial relationships that could be construed as a potential conflict of interest.
